# The therapeutic potential of induced hepatocyte-like cells generated by direct reprogramming on hepatic fibrosis

**DOI:** 10.1186/s13287-018-1127-3

**Published:** 2019-01-11

**Authors:** Suhyun Park, Seon In Hwang, Jonghun Kim, Seoyeon Hwang, Sohee Kang, Sera Yang, Jonghwa Kim, Wonseok Kang, Kyun-Hwan Kim, Dong Wook Han, Yong-Han Paik

**Affiliations:** 1 Department of Medicine, Samsung Medical Center, Sungkyunkwan University School of Medicine, 81 Irwon-Ro, Gangnam-Gu, Seoul, 06351 South Korea; 2 Department of Health Sciences and Technology, SAIHST, Sungkyunkwan University, Seoul, South Korea; 3 Department of Stem Cell Biology, School of Medicine, Konkuk University, Seoul, South Korea; 4 Department of Advanced Translational Medicine, School of Medicine, Konkuk University, Seoul, South Korea; 5 Konkuk University Open Innovation Center, Research Institute of Medical Sciences, Konkuk University, Seoul, South Korea; 6 Department of Pharmacology, Center for Cancer Research and Diagnostic Medicine, IBST, School of Medicine, Konkuk University, Seoul, South Korea

**Keywords:** Animal models, Disease modeling, CCl_4_, Stem cell transplantation, Hepatic fibrosis, Cell and tissue-based therapy, Liver regeneration, Induced pluripotent stem cells, Hepatic stem cells, Hepatic disorders, Small molecule based reprogramming, Direct reprogramming, Stem cell therapy

## Abstract

**Background:**

Until now, there is no effective anti-fibrotic therapy available for liver cirrhosis. Stem cell therapies have been studied for the treatment of hepatic fibrosis. However, the use of embryonic stem cells or induced pluripotent stem cells (iPSC) has limitations such as ethical concern or malignancy potential. Induced hepatocyte-like cells (iHEPs) generated by direct reprogramming technology may overcome these limitations.

**Methods:**

In this study, we generated iHEPs by direct reprogramming from mouse embryonic fibroblast (MEF) either using specific transcription factors such as c-Myc and Klf-4 (type A), or adding small molecules to HNF1α (type B).

**Results:**

We investigated the effect of iHEPs on acute liver injury and chronic hepatic fibrosis animal models induced by CCl_4_ intra-peritoneal injection in BALB/C nude mice. In acute liver injury model, serum AST/ALT levels peaked at 24 h after CCl_4_ injection. Intra-splenic transplantation of iHEPs significantly attenuated CCl_4_-induced acute liver injury. GFP-labeled iHEPs (type A) migrated to the liver after intra-splenic transplantation that was confirmed by Western blotting and immunofluorescence staining. We found that GFP and albumin were co-localized in migrated iHEPs in the liver suggesting migrated iHEPs were functional. In chronic hepatic fibrosis mice experiment, transplantation of either type A or type B iHEPs significantly attenuated liver fibrosis induced by CCl_4_ injection for 10 weeks.

**Conclusions:**

Our study suggests that iHEPs may be used as a novel therapeutic strategy for the treatment of hepatic fibrosis.

**Electronic supplementary material:**

The online version of this article (10.1186/s13287-018-1127-3) contains supplementary material, which is available to authorized users.

## Background

Liver cirrhosis represents the main pathological outcome for the majority of chronic liver diseases [1, 2]. The patients with advanced liver cirrhosis may die from serious complications including ascites, hepatic encephalopathy, and variceal hemorrhage. Liver is a highly regenerative organ with a remarkable ability to restore its original mass following parenchymal cell loss [3]. It also performs multiple functions including metabolic activities, homeostasis, nutrition, and immune defense. Liver fibrosis is a common pathophysiology resulting in liver cirrhosis that is the final stage of chronic liver diseases. In patients with advanced liver cirrhosis, excessive collagen production from activated myofibroblasts interrupts hepatic blood flow, and parenchymal functional reserve decreased [4–6]. Until now, there is no effective anti-fibrotic therapy for liver cirrhosis. Liver transplantation is the only available treatment for advanced liver cirrhosis; however, it has limitations including donor organ shortage, immunological rejection, side effects of drug, and high cost [7].

Because of these reasons, cell-based therapy including stem cell therapy [8–11] has been considered as a potential therapy for liver cirrhosis. Yamanaka group reported that they succeeded to generate the induced pluripotent stem cells (iPSCs) from mouse or human fibroblasts by defined factors [12]. Functional hepatocytes can be generated from mouse iPSCs [13]. Moreover, transplantation of human iPSC-derived hepatocyte-like cells (iPSC-HLCs) successfully ameliorated lethal acute liver injury induced by CCl_4_ and rescued mice from acute liver failure [14]. Therefore, iPSC-HLCs have a therapeutic potential for liver failure and end stage liver disease in human. However, iPSCs have several critical limitations including low production efficiency and potential tumorigenicity for the clinical application in human [15].

Recently, investigators directly reprogrammed mouse embryonic and adult fibroblasts to hepatocyte-like cells by introduction of transcription factors. These cells called induced hepatocyte-like cells (iHEPs) that have multiple hepatocyte-specific features and reconstitute damaged liver tissues after transplantation [16]. This innovative technology will enable us to produce hepatocyte-like cells more efficiently without a requirement of embryonic stem cells (ESCs) or iPSCs. In this study, we aimed to investigate the therapeutic potential of iHEPs transplantation on hepatic fibrosis induced by intra-peritoneal CCl_4_ injections. In this study, we used iHEPs generated by two different direct reprogramming techniques [17]. One direct reprogramming techniques was retroviral introduction of specific transcription factors such as c-Myc and Klf-4 (type A), and the other was by adding small molecules to specific transcription factor HNF1α (type B) [17]. Transplantation of iHEPs generated by two different techniques significantly attenuated liver fibrosis induced by CCl_4_ injections. Our study suggests that iHEPs may be used as a novel therapeutic strategy for treatment of hepatic fibrosis.

## Methods

### Reagents

CCl_4_ (carbon tetrachloride) was purchased from MERCK (KGaA, 64271 Darmstadt, Germany). Olive oil was purchased from Sigma-Aldrich (Sigma-Aldrich, St. Louis, MO, USA).

### Generation of induced hepatocyte-like cells (iHEPs) by direct reprogramming

iHEPs were generated by two different direct reprogramming techniques. One was generated by retroviral introduction of specific transcription factors including c-Myc and Klf-4 with FOXA3 and HNF4α (type A), the other was generated by small molecules such as A-83-01, CHIR-99021, and BMP-4 (type B) with HNF1α [17]. To generate mouse iHEPs, fibroblasts (5 × 10^4^ cells) were infected with pMX retrovirus expressing the transcription factors in different combinations for 48 h. Prior to fibroblast transduction, the viral particles were produced for 48 h after transfecting 293FT cells using a single pMX retroviral vector coding for one gene, together with the packaging plasmid pCL-Eco as previously described [18, 19]. The viral batches that showed transduction efficiency higher than 80% using the control GFP retrovirus were first transduced to generate iHEPs. Cells transduced with different combinations of factors were cultured in standard hepatocyte culture medium (HCM): DMEM/F-12 supplemented with 10% FBS (Biowest), 0.1 μM dexamethasone (Sigma), 10 mM nicotinamide (Sigma), 1% ITS (insulin-transferrin-selenium) premix (Gibco), and penicillin/streptomycin/glutamine (Invitrogen), 10 ng/ml, FGF4 (fibroblast growth factor 4, Peprotech), and both HGF (hepatocyte growth factor, Peprotech) and EGF (Epidermal growth factor, Peprotech) at a final concentration of 20 ng/ml on a type I collagen-coated dish (Sigma) in the presence or absence of distinct small molecules, 2 μM A-83-01 (Tocris), 3 μM CHIR99021 (Enzo Life Sdciences), and/or 10 ng/ml BMP4 (Peprotech). Details about generation of induced hepatocytes are shown in the reference [17, 20].

### Mice

Male BALB/C nude mice, 6 weeks old, were obtained from Orient Bio (Seongnam, Korea). Mice were handled at the institute’s (Samsung Medical Center Laboratory Animal Research Center, Seoul, Korea) animal facility, and all treatments were in accordance with the legal and institutional guidelines.

### Experiment of acute liver injury

**Fig. 1:**
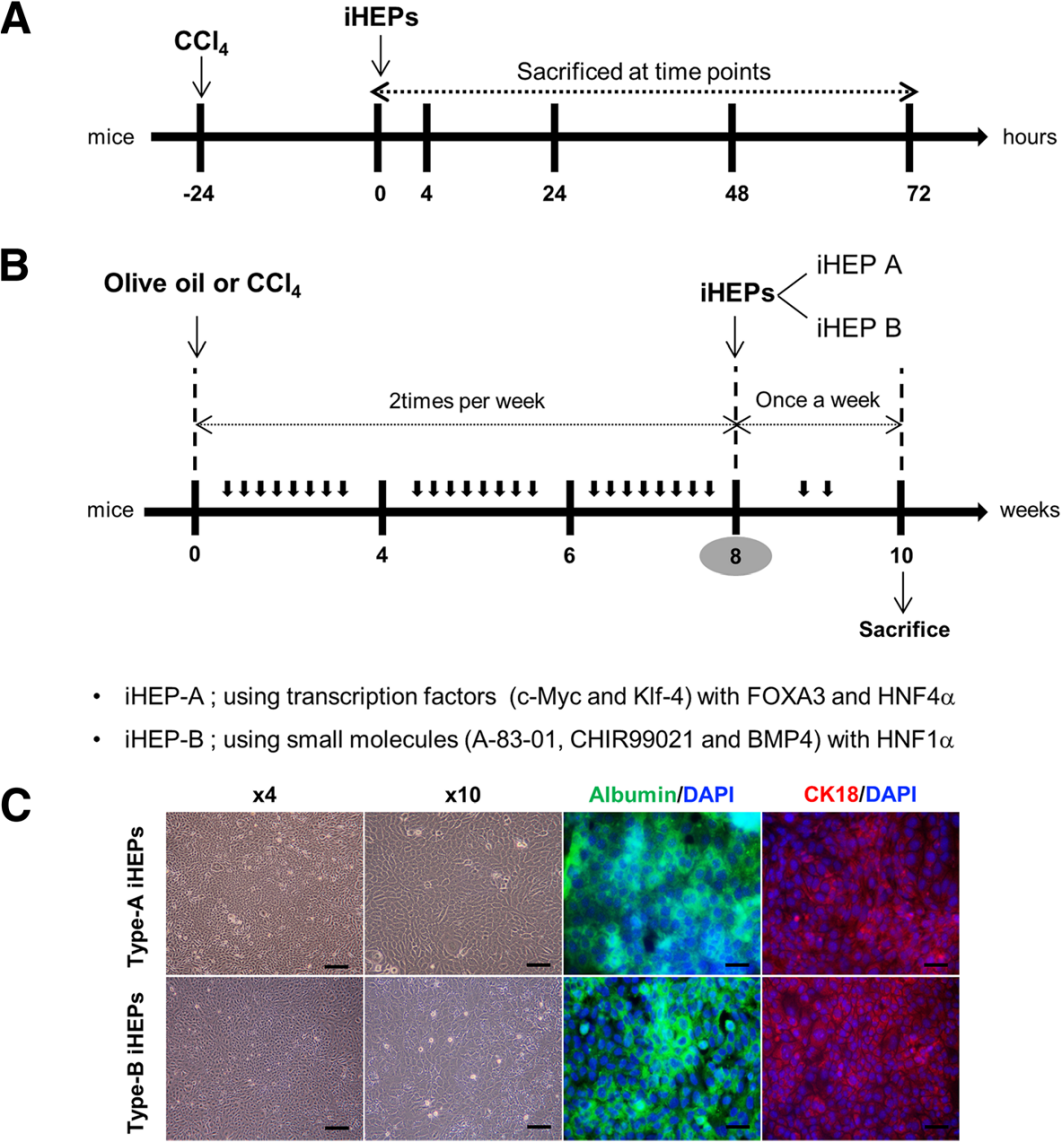
Experimental design for mouse models of acute liver injury and chronic hepatic fibrosis. Eight weeks of BALB/C nude mice were used in both acute and chronic liver injury models. **a** In acute liver injury model, single CCl_4_ dissolved in olive oil (0.5 μl/g) were administrated by intra-peritoneal injection. Intra-splenic iHEPs transplantation was processed at 24 h after administration of CCl_4_. Mice were sacrificed at specific time points. **b** In chronic hepatic fibrosis model, CCl_4_ (0.25 μl/g) or olive oil was intra-peritoneally injected twice a week during 8 weeks. In CCl_4_ + iHEPs group, iHEPs (1 × 10^6^ cells/100 μl, either type A or type B) were transplanted into spleen at the end of 8 weeks of CCl_4_ injections. After a total of 10 weeks of CCl_4_ treatment, all mice were sacrificed for analysis. **c** Immunofluoresence staining for characterization of induced hepatocyte-like cells (types A and type B) DAPI, nuclear staining; 488-Green, anti-albumin staining, 594-Red, anti-CK18; scale bar, 100 μm

For acute liver injury experiment, a single intra-peritoneal CCl_4_ (0.5 μl/g) mixed with olive oil (1:3 volume) injection was performed to 8 weeks of male of BALB/C nude mice. A total of 1 × 10^6^ iHEPs (type A) were re-suspended in 100 μl DMEM (without FBS and antibiotics) and transplanted through spleen injection [17, 21–23]. In the light of CCl_4_ toxicity, iHEPs transplantation were processed at 24 h after administration of CCl_4_ to avoid cell damage by CCl_4_ toxicity [24–27]. Mice were randomly assigned to five groups according to time points: 0, 4, 24, 48, and 72 h (from 24 h of post-injection CCl_4_) (Fig. 1a). Following transplantation in acute liver injury model, samples were collected at specific time points above mentioned.

### Experiment of hepatic fibrosis

For experiment of chronic hepatic fibrosis models, mice were randomly assigned to four groups: Control group, CCl_4_ group, CCl_4_ + iHEPs type A group, and CCl_4_ + iHEPs type B group. In control group, olive oil was intra-peritoneally injected twice a week during 8 weeks. In CCl_4_ group, CCl_4_ (0.25 μl/g) mixed with olive oil (1:3 volume) was intra-peritoneally injected twice a week during 8 weeks. In CCl_4_ + iHEPs group, at the end of 8 weeks of CCl_4_ injection, iHEPs (1 × 10^6^ cells/100 μl) mixed with free DMEM (−/−, no FBS, no antibiotics) were injected into spleen at 24 h after the last CCl_4_ injection. The previous study reported that iHEPs (types A and B) represent functional hepatic cells and iHEPs share molecular and functional characteristics with primary hepatocytes [17]. Based on this reported study, we used two kinds of iHEPs generated by different techniques (Fig. 1). One was generated by retroviral introduction of specific transcription factors including c-Myc and Klf-4 with FOXA3 and HNF4α (type A), the other was generated by small molecules such as A-83-01, CHIR-99021, and BMP-4 to HNF1α (type B) [17]. After intra-splenic iHEPs transplantation, CCl_4_ (0.5 μl/g) were injected once a week to maintain liver injury for 2 weeks. After a total of 10 weeks of CCl_4_ treatment, all mice were sacrificed for analysis.

### Analysis of serum AST and ALT

Serum levels of aspartate aminotransferases (AST) and alanine aminotransferases (ALT) were assayed with colorimetric slides (Fuji Dri-Chem Slide GPT/AST-PIII and Fuji Dri-Chem Slide GPT/ALT-PIII; Fuji, Japan) according to instructions given by the manufacturer.

### Protein extraction

Proteins were extracted from liver tissue by using T-PER lysis buffer (Sigma-Aldrich, St. Louis, MO, USA) dissolved with proteasome and phosphatase inhibitors, respectively. Protein concentrations were assessed through the BCA assay (Thermo Fisher Scientific Korea Ltd.) according to the protocol recommended by the manufacturer.

### Western blot analysis

Twenty micrograms of protein was separated on NuPAGE Novex Bis–Tris 4–12% gels (Invitrogen) and electroblotted onto PVDF membranes. Membranes were then incubated in blocking solutions (5% non-fat milk in 20 mM Tris-HCl, 150 mM NaCl, and 0.1% Tween-20), followed by overnight incubation with a mouse monoclonal anti-alpha GFP 1:1000 (Applied Biological Materials, Viking Way, Richmond BC, Canada V6V 2G2). Peroxidase-labeled anti-mouse IgG diluted 1:1000 (Cell Signaling Technology, Danvers, MA, USA) were used as a secondary antibody. Bound peroxidase activity was revealed using Super-Signal West Pico Chemiluminescent Substrate (Pierce, Rockford, IL, USA).

### Immunocytochemistry

For immunofluorescence staining, the cells were fixed with 4% paraformaldehyde (Biosesang) for 10 min at room temperature, and then blocked with DPBS (Welgene) containing 0.3% Triton X-100 (Sigma) and 5% FBS (Gibco) for 1 h at room temperature. The cells were then incubated with primary antibodies overnight at room temperature in dark moist chamber, washed three times with DPBS, and then incubated with appropriate fluorescence-conjugated secondary antibody for 1 h at room temperature in the dark moist chamber. Nuclei were stained with DAPI. Cells were observed under a fluorescence microscope or a confocal laser scanning microscope (Carl Zeiss Co., zeiss lsm 700 confocal microscopy). Primary antibodies used for immunofluorescence are as follows: mouse anti-Albumin (R&D Systems, 1:100) and mouse anti-CK18 (Abcam, 1:200) [17].

### Immunofluorescence staining and immunohistochemistry

For immunofluorescence antibody staining, liver samples were maintained in 4% para-formaldehyde during overnight. After then, samples were incubated in 10% sucrose during 2 h and 30% sucrose during overnight prepared as frozen sections. The livers were dissected into pieces, immediately incubated with an optimal cutting temperature compound (OCT compound, Sakura Fine technical, USA, Inc.) and held in liquid nitrogen for 20 to 30 s. The frozen liver sections were directly transferred to a − 70 °C freezer refrigerator. Cryostat slides sections of liver tissue excised from the mice were stained for observing GFP-expressing cells in the liver tissue and expression of albumin in the liver tissue. The frozen sections were allowed to melt in cold PBS (no detergent) for 5 min and placed on slides. Blocking for 1 h at room temperature with goat serum diluted in PBS. Add diluted primary antibody [anti-albumin (Bethyl Laboratories, A90-134A, Mouse albumin antibody)] into goat serum with PBS. Incubate in a moist chamber during overnight at 4 °C. Wash slides in cold PBS to remove excess anti-serum at room temperature. Add diluted goat anti-mouse lgG-Alexa Fluor 594 as second antibody. Incubate in a moist chamber for 1 h at room temperature and mount with fluorescence mounting medium with DAPI and seal coverslip.

For immunohistochemistry, liver tissues were collected after sacrifice and fixed using 10% formalin solution for 24 h and paraffin-embedded. Paraffin slide sections of liver tissue excised from the mice were stained with hematoxylin and eosin (HE), picro-sirius red staining, and Massons’ trichrome staining and processed for routine histological examination. The EnVisionTM System (DAKO, Carpinteria, CA, USA) was used for IHC staining, and the staining was according to the protocol recommended by the manufacturer. Sections (4 μm) were cut, de-waxed and then rehydrated and subjected to antigen retrieval using target retrieval solution (citrate pH 6; DAKO Korea). Reducing nonspecific background staining using protein block serum-free solution (X0909, DAKO Korea), the sections were incubated overnight at 4 °C with primary antibodies in antibody diluents (S3022, DAKO Korea) against anti-alpha GFP (ABM, G096, 1:500). After washing three times, slides were incubated with secondary antibody (Fluoresence, Alexa, 488, 594) in antibody diluents for 1 h at room temperature. Then, DAPI staining for 5 min and washing three times and mounting slides by using mounting solution for 24 h at room temperature. The slide images were photographed using Image Scan Scope AT.

### Quantification of necrosis area

To identify the necrosis area of acute liver injury induced by CCl_4_, the liver necrosis area was stained with hematoxylin and eosin staining and then quantified by Image J which is open source image processing program designed for scientific multidimensional images.

### Hematoxylin and eosin staining

To identify the damage of acute liver injury induced by CCl_4_, the liver damaged area was stained with hematoxylin and eosin. H&E staining according to the manufacturer’s instructions (Hematoxylin and Eosin stain kit, VECTOR Laboratories, H-3502).

### Sirius red staining

To identify the extent of liver fibrosis induced by CCl_4_, the liver fibrosis area was stained with Sirius red and then quantified by Image J which is open source image processing program designed for scientific multidimensional images. In brief, liver tissue sections were incubated in picro-sirius red (sigma, USA) for 30 min and washed with acetic acid and water.

### Masson’s trichrome staining

To identify the extent of liver fibrosis induced by CCl_4_, the liver fibrosis area was stained with Masson’s trichrome staining and then quantified by Image J which is open source image processing program designed for scientific multidimensional images. Paraffin slides were prepared as mentioned above and subjected to Masson’s trichrome staining according to the manufacturer’s instructions (Trichrome Stain Masson Kit, Sigma-Aldrich, HT15).

### Quantitative real-time PCR

Total RNA from liver tissue slices was extracted using the RNeasy Mini Kit (Qiagen, Hilden, Germany) according to the manufacturer’s instructions. The RNA concentrations were measured on a NanoDrop spectrophotometer (ND-2000). cDNA was synthesized using High-Capacity RNA-to-cDNA™ Kit (ThermoFisher Scientific, 4387406) in a volume of 20 μl. cDNA was diluted to 5 ng/μl and 2 μl/reaction (10 ng) and then used for qRT-PCR analysis. PCR reactions were performed in a 10 μl reaction volume containing qPCR master mix (AccuPower ® 2X GreenStar™ qPCR Master Mix). qRT-PCR reactions were performed on a ABI7900HT thermal cycler (Applied Biosystems, Foster City, CA, USA). Relative gene expression was calculated using the 2^-ΔΔCt^ method with GAPDH as housekeeping gene (see in Additional file 1: Table S1).

### Statistical analysis

The data were expressed as the mean ± S.E.M. (standard error of the mean). Statistical analyses were undertaken using GraphPad prism version 5.01 (GraphPad Software, San Diego, CA, USA). Data were analyzed using one-way analysis of variance (ANOVA) or analyzed by the Student *t* test. Data were considered to be statistically significant when *p* value < 0.05.

## Results

### Transplantation of iHEPs significantly attenuated CCl_4_-induced acute liver injury in BALB/C nude mice

**Fig. 2:**
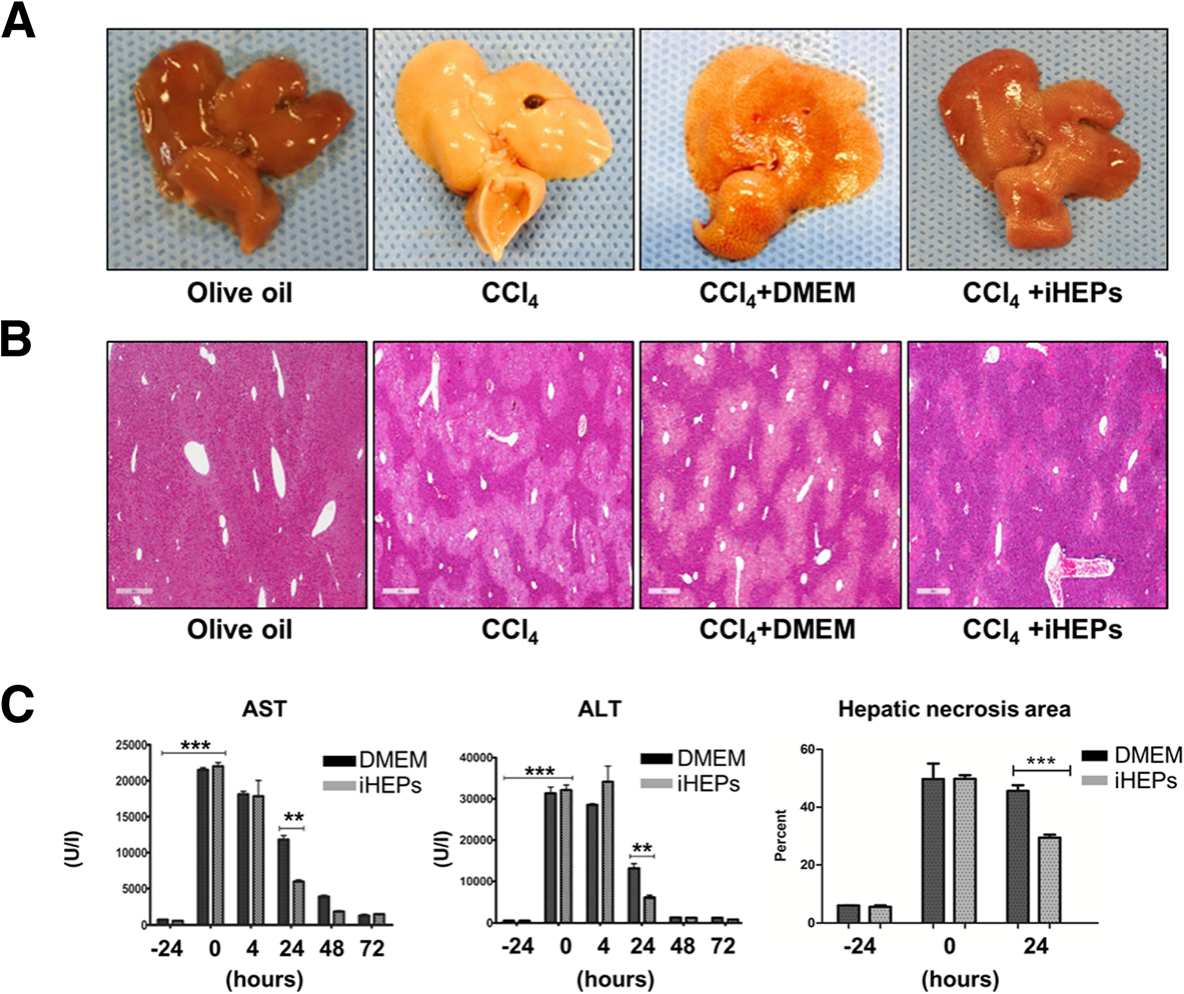
CCl_4_-induced liver acute injury was significantly attenuated by iHEPs transplantation in BALB/C nude mouse. **a** Representative of gross finding of the liver in acute liver injury model by single intra-peritoneal CCl_4_ injection. A total of 1 × 10^6^ iHEPs (type A) or DMEM only were transplanted through intra-splenic injection. Left to right: olive oil, CCl_4_, CCl_4_ + DMEM, and CCl_4_ + iHEPs group, respectively. **b** Representative histological finding (H&E staining) of the liver (scale bar, 400 μm). **c** Serum levels of AST/ALT and hepatic necrosis area in acute liver injury models at indicated time points. Black colored bar indicates intra-splenic DMEM injection only group, gray-colored bar represents intra-splenic iHEPs transplantation group (***P < 0.001, **P < 0.01)

Through the immunofluorescence staining with iHEPs (types A and B), we confirmed that our iHEPs represent functional features as hepatic cells (Fig. 1c). Therefore, to identify the potential therapeutic effects of iHEPs, we induced acute liver injury by single intra-peritoneal CCl_4_ injection in BALB/C nude mice. As expected, gross finding showed that CCl_4_ induced yellowish color change, swelling, hard texture, and rough surface of the liver compared with those of control mouse which exhibited soft texture and smooth surface (Fig. 2a). Furthermore, H&E staining of liver sections from the iHEPs transplanted mice demonstrated only moderate necrosis involving the centrilobular areas, maintaining relatively normal acinar structure compared with CCl_4_ group or CCl_4_ + DMEM group (Fig. 2b). To assess the degree of acute liver injury induced by CCl_4_ injection, we measured serum AST/ALT and hepatic necrosis area (Fig. 2c). A single peritoneal injection of CCl_4_ increased in serum aspartate aminotransferase (AST) and alanine aminotransferase (ALT) levels which were significantly attenuated in iHEPs transplantation group, as well as hepatic necrosis area, but not in control (DMEM only transplantation) group at 24 h after transplantation (Fig. 2c, **P < 0.01, ***P < 0.001). These results indicate intra-splenic transplantation of iHEPs significantly attenuated CCl_4_-induced acute liver injury both in histology and serology.

### Intra-splenic transplanted iHEPs migrated to the liver and functioned as albumin-producing cells

**Fig. 3:**
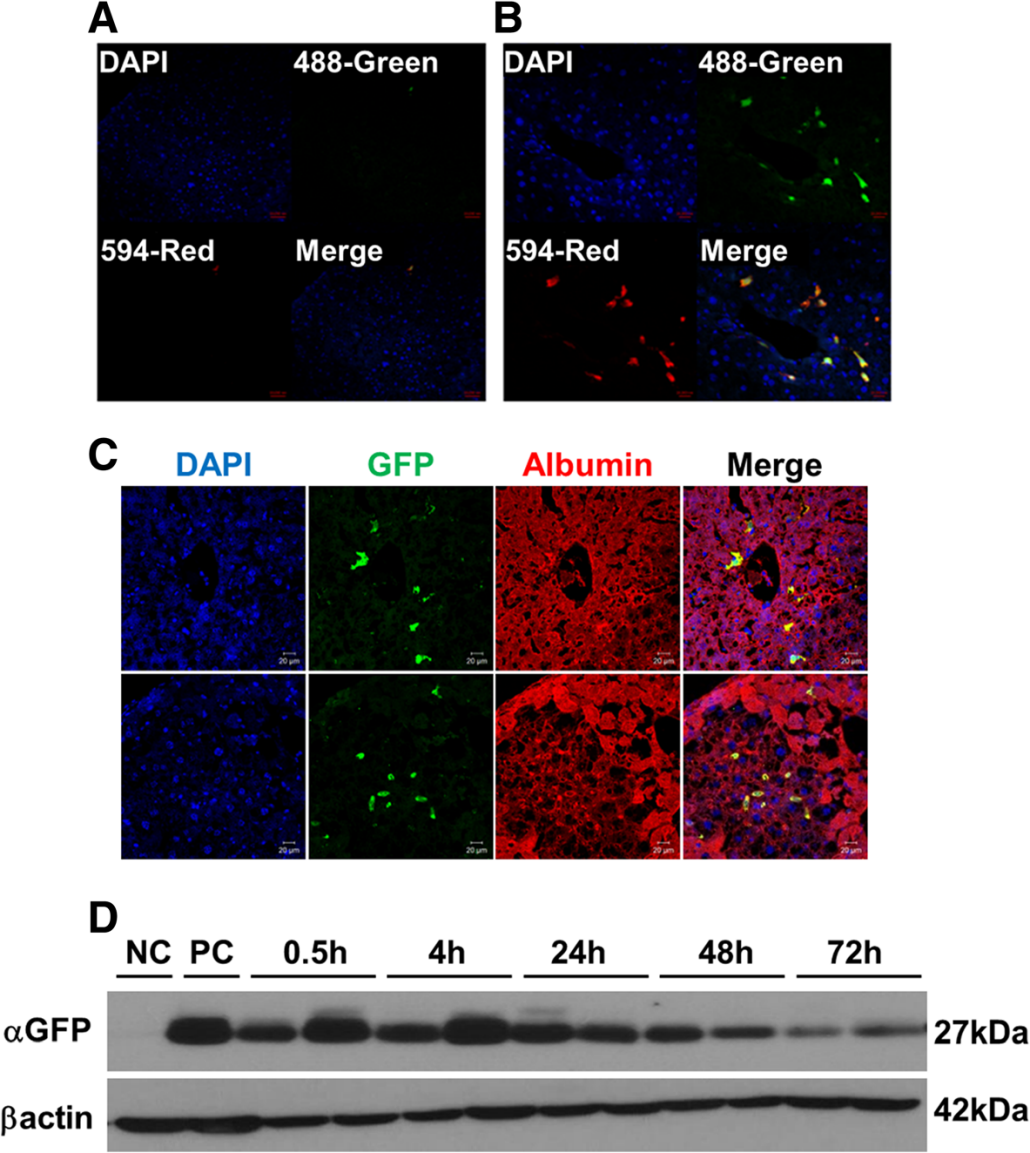
Identification of migration and function of intra-splenically transplanted iHEPs in acute liver injury model. The migration of intra-splenically transplanted iHEPs into the liver was investigated using alpha-GFP-labeled iHEPs. **a** Immunofluorescence staining of liver after 4 h of DMEM intra-splenic injection. **b** iHEPs intra-splenic transplantation. DAPI, nuclear staining; 488-Green, GFP-labeled iHEPs-type A fluorescence; 594-Red, anti-GFP immunofluorescence staining; Merge, merged image of 488-Green and 594-Red. Scale bar, **a**, 50 μm; **b**, 20 μm. **c** Upper and lower rows represent immunofluorescence staining in two different sites of liver sections. DAPI, nuclear staining; GFP, GFP fluorescence; Albumin, anti-albumin staining; Merge, merged image of GFP and albumin staining. Scale bar, 20 μm. **d** Western blotting for GFP or β-actin from mouse liver tissue sacrificed at indicated time points. NC, negative control; PC, positive control (iHEPs)

We confirmed that intra-splenic transplantation of iHEPs attenuated acute liver injury. We hypothesized that transplanted iHEPs migrated and functioned in the liver. To identify the migration of iHEPs, we transfected alpha-GFP into iHEPs using lentiviral vector. Through immunofluorescence staining, no GFP-labeled cells were detected in control group (DMEM injection only) at 4 h after transplantation (Fig. 3a), but GFP-labeled iHEPs were detected around portal tracts at 4 h after transplantation by GFP fluorescence (488-Green) and by anti-GFP staining (594-Red) (Fig. 3b), demonstrating that transplanted iHEPs migrated into the liver. Moreover, we found that engrafted iHEPs function as primary hepatocyte through observation of co-localization of albumin and GFP-labeled iHEPs by immunofluorescence staining (Fig. 3c). Furthermore, we confirmed that engraftment of iHEPs remained stable in the liver during 72 h after transplantation (Fig. 3d). These data indicated that transplanted iHEPs migrate into the liver and function as primary hepatocyte.

### Transplantation of iHEPs significantly decreased CCl_4_-induced liver fibrosis in BALB/C nude mice

**Fig. 4:**
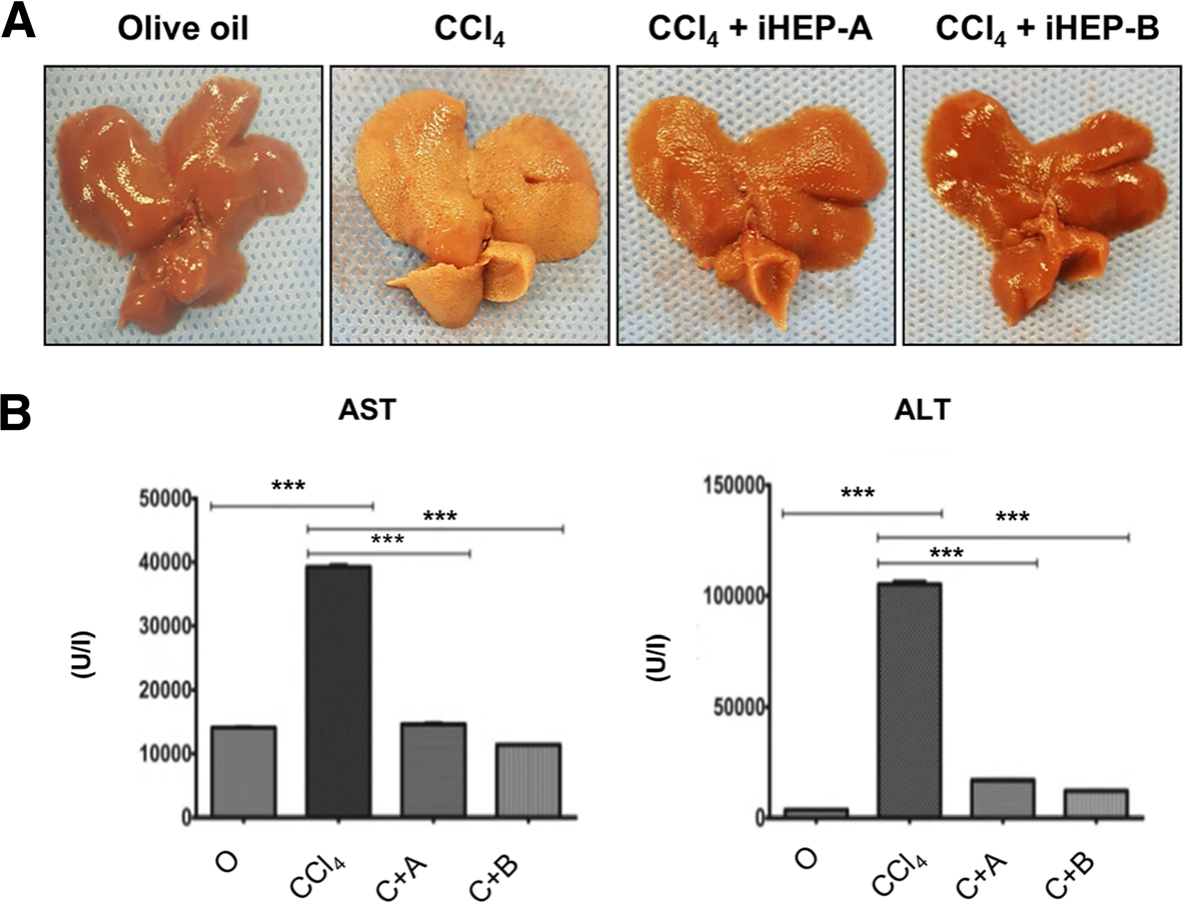
CCl_4_-induced chronic hepatic fibrosis was significantly attenuated by iHEPs transplantation in BALB/C nude mouse. **a** Representative of gross finding of the liver in chronic hepatic fibrosis model by intra-peritoneal CCl_4_ injections for 10 weeks. A total of 1 × 10^6^ iHEPs (type A or type B) or DMEM only were transplanted through intra-splenic injection. Left to right: olive oil, CCl_4_, CCl_4_ + iHEPs (type A), and CCl_4_ + iHEPs (type B), respectively. **b** Serum levels of AST and ALT after 10 weeks of CCl_4_ injections in chronic hepatic fibrosis model (***P < 0.0001)

We next evaluated the therapeutic potential of iHEPs transplantation in chronic hepatic fibrosis developed by chronic CCl_4_ injections. We used two types of iHEPs generated by two different techniques: Type A iHEPs (using transcription factors) and type B iHEPs (using small molecules). As expected, total 10 weeks of CCl_4_ injections established significant liver fibrosis. The gross finding of liver indicated that chronic CCl_4_-treated mice liver showed yellowish color change, nodular surface, and firm texture as compared to control (olive oil) group. In contrast, CCl_4_-treated mice liver that was transplanted with either type A or type B iHEPs showed little color change, smooth surface, and soft texture similar to control group (Fig. 4a). To evaluate the extent of hepatic dysfunction, we measured serum AST and ALT. As shown in Fig. 4b, both AST and ALT levels were highly elevated in CCl_4_-treated mice as compared to control (olive oil) group. iHEPs transplantation (either iHEPs type A or type B) significantly decreased AST and ALT levels even after 10 weeks of CCl_4_ injections (Fig. 4b).

**Fig. 5:**
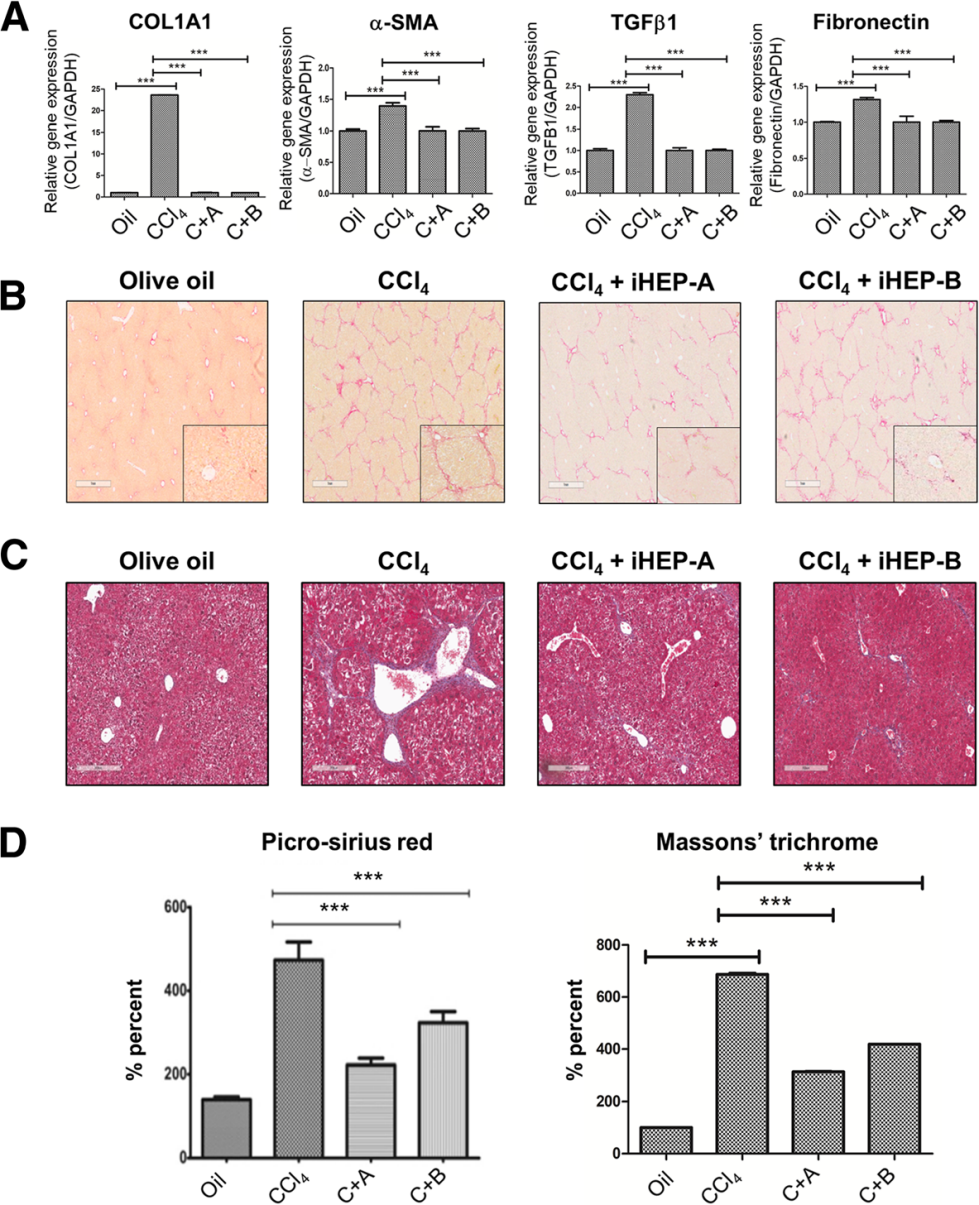
Anti-fibrotic effect of intra-splenic transplanted iHEPs on CCl_4_-induced chronic hepatic fibrosis in BALB/C nude mice. **a** CCl_4_ induced expression of fibrotic markers in chronic hepatic fibrosis models of BALB/C nude mice. mRNA expression of COL1A1, a-SMA, TGFb1 and fibronectin, respectively (***P < 0.0001). **b** Representative histological findings of liver sections that were stained with Sirius red for the assessment collagen contents. Deep red staining represents collagen deposition. Scale bar, 1 mm. **c** Representative histological findings of liver section that were stained with Masson’s trichrome staining for the assessment of collagen contents. Deep blue staining represents collagen deposition. Scale bar, 300 μm. **d** Quantification of collagen contents through image analysis of sirius red stained (Left) and Masson’s trichrome stained (Right) area using Image J software (***P < 0.0001, C + A; CCl_4_ + iHEPs-A, C + B; CCl_4_ + iHEPs-B)

Then, we evaluated mRNA expression levels in CCl_4_-induced chronic hepatic fibrosis tissue through qRT-PCR. Predictably, CCl_4_-induced significant increase of mRNA expression of fibrotic markers such as COL1α1, α-SMA, TGFβ1, and fibronectin and those expression levels were significantly reduced in iHEPs transplantation groups (Fig. 5a). To identify the extent of hepatic fibrosis in liver tissue, we performed collagen staining by sirius red and Massons’ trichrome staining. As we expected, 10 weeks of CCl_4_ injections induced a significant increase in collagen contents in mouse liver. Importantly, chronic CCl_4_-treated mice liver that was transplanted with either iHEPs type A or B showed significantly decreased hepatic fibrosis compared CCl_4_-treated mice (Fig. 5b, c). Furthermore, to identify and quantify the collagen deposition, we performed image analysis of sirius red stained and Massons’ stained area, respectively (Fig. 5d). These data clearly demonstrated a therapeutic potential of iHEPs generated by two different techniques on hepatic fibrosis or liver cirrhosis.

### Investigation of potential mechanisms on iHEPs in hepatic disease models

To investigate potential mechanisms of iHEPs-induced anti-fibrotic effect in our animal experiments, we evaluated mRNA expression for inflammatory cytokines and anti-oxidant molecules (see in Additional file 2: Figure S1). mRNA expression levels of pro-inflammatory cytokines such as TNFα, IL-6, and IL-1β were significantly decreased in iHEPs transplantation groups compared to CCl_4_ only group. Moreover, anti-oxidant target genes such as NQO-1, HO-1, SOD-1, CAT, and GST with its transcription factor NRF2 mRNA levels were significantly increased in both iHEPs transplantation groups compared to CCl_4_ only group. These data indicate that iHEPs-mediated anti-inflammatory and anti-oxidant effect may at least in part contribute to anti-fibrotic effect.

## Discussion

This study aimed to explore the therapeutic potential of induced hepatocyte-like cells (iHEPs) generated through two different direct reprogramming techniques from mouse fibroblasts on chronic CCl_4_-induced hepatic fibrosis. Recently, considerable progress has been established in stem cell research, especially cell therapy with hematopoietic stem cells and iPSCs. Preclinical study reported that infusion of bone marrow-derived cells or hematopoietic stem cells can reduce hepatic fibrosis [28, 29]. Small-scale clinical study reported that infusion of bone marrow-derived stem cells accelerate liver regeneration and improve liver function in patients with liver cirrhosis [30]. However, these effects are not universally reproduced and treatment effects are not always durable [31]. Moreover, recent REALISTIC trial reported no differences in MELD (Model for End-stage Liver disease) score change between G-CSF plus CD133-positive hematopoietic stem cell group and standard care group in patients with liver cirrhosis [31].

iPSCs have a potential to self-replicate and differentiate into hepatocyte-like cells; therefore, iPSCs would be an attractive cell source to treat hepatic fibrosis or liver cirrhosis. Several groups demonstrated that iPSC-HLC cell therapy was therapeutically effective in mice models of liver failure [14, 32]. However, safety concerns including teratoma formation and tumor generation still limit clinical application of iPSC-HLC in the treatment of liver diseases.

Recently, by direct reprogramming of fibroblasts into multiple adult cell types including neurons [18], cardiomyocytes [33], and hepatocytes [16, 20] through expression of specific transcription factors has been reported. Investigators directly converted mouse embryonic and adult fibroblasts to hepatocyte-like cells by introduction of transcription factors such as Hnf4α plus FOXA1, FOXA2, or FOXA3 by the combination of GATA4, Hnf1α, and FOXA3 and inactivation of p19. These cells called induced hepatocyte-like cells (iHEPs) have multiple hepatocyte-specific features and reconstitute damaged liver tissues after transplantation [16, 20].

In this study, we used iHEPs by adopting two different direct reprogramming techniques. One is generated by retroviral introduction of specific transcription factors c-Myc and Klf-4 and the other is by using small molecules A-83-01, CHIR-99021, and BMP-4 [17]. These two technologies have several advantages and limitations. First, we can infinitely expand the cell population and autologous transplantation is possible. Second, c-Myc and Klf-4 can accelerate conversion kinetics, resulting in dramatically improved iHEPs generation efficiency. However, c-Myc and Klf-4 have oncogenic properties. These two factors should be excluded for future clinical applications. For these reasons, we replace c-Myc and Klf-4 with small molecules for generating advanced iHEPs (iHEPs-type B) and performed our experiments by using this cell line as parallel. Collectively, through this technology, we can get more mature and high yield iHEPs from mouse fibroblasts without potential risk of tumorigenicity [17, 34, 35]. We confirmed that intra-splenic transplanted iHEPs migrated to the liver and functioned like as primary mature hepatocyte. In the acute liver injury mice model, iHEPs transplantation significantly diminished the extent of acute liver injury in BALB/C nude mice. Moreover, we confirmed that transplantation of iHEPs significantly attenuated chronic CCl_4_-induced liver fibrosis in mice. Furthermore, we confirmed that engrafted iHEPs in the liver co-localize with albumin suggesting these iHEPs possess functional characteristics of primary hepatocytes that can restore liver function and induce liver regeneration. Therefore, we think that the function of engrafted iHEPs as primary hepatocytes importantly contribute to anti-fibrotic effect in the fibrotic liver.

Recently, Song et al. reported that induced hepatocytes that were generated by direct reprogramming from hepatic myofibroblasts attenuate liver fibrosis [35]. They used lentiviral or adenoviral expression of transcriptional factors FOXA3, GATA4, HNF1α, and HNF4α to generate iHEPs from profibrogenic myofibroblasts. These results corroborate our results that iHEPs generated by direct reprogramming show functional benefits and therefore have therapeutic potential in the treatment of hepatic fibrosis and liver cirrhosis.

Our data demonstrated that iHEPs have a therapeutic potential in in vivo models such as acute liver injury and chronic liver fibrosis induced by CCl_4_. However, understanding of iHEPs mechanical association in the liver is still largely unknown. In this regard, previous study reported that chronic liver inflammation leads to fibrosis and cirrhosis [36]. To investigate potential mechanisms of iHEPs-induced anti-fibrotic effect in our animal experiments, we measured inflammatory cytokines such as IL-1β, IL-6, and TNFα (Additional file 2: Figure S1A–C). As expected, mRNA expression levels were significantly decreased in both iHEPs transplantation groups compared to CCl_4_ only group. These results suggest a potential anti-inflammatory effect of iHEPs.

Oxidative stress is a key pathogenic mechanism for hepatic fibrogenesis. NRF2 (Nuclear factor erythroid-derived 2-like 2) is transcription factor that regulates the expression of anti-oxidant proteins and protects against oxidative damage triggered by liver injury and inflammation [37, 38]. Based on these reports, we measured NRF2 and its downstream anti-oxidant molecules. Our data showed that mRNA expressions of NRF2 and its target genes were upregulated in both iHEPs transplantation groups compared to CCl_4_ only group. These data suggest that iHEPs might have an anti-oxidant effect resulting in antifibrotic effect. However, the underlying mechanisms of iHEPs-induced antifibrotic effect remains to be fully elucidated in future study.

Our study clearly demonstrated iHEPs transplantation attenuated acute liver injury and decreased hepatic fibrosis induced by CCl_4_ injections. However, previous studies adopting hematopoietic stem cell transplantation showed anti-fibrotic effect in rodent model of fibrosis; however, it failed to demonstrate anti-fibrotic effect in human clinical trial. Fibrosis is qualitatively and quantitatively different in rodent models. Hepatic fibrosis in mouse has less collagen cross-linking and more spontaneous remodeling than is typically seen in humans [39]. Further clinical study warrants to prove the therapeutic effect of iHEPs transplantation in patients with advanced hepatic fibrosis or liver cirrhosis.

## Conclusion

Our study demonstrated that induced hepatocyte-like cells generated by two different techniques migrated and functioned in the liver and attenuated liver injury and hepatic fibrosis in mice. These results suggest that iHEPs transplantation may serve as a novel therapeutic strategy for treatment of liver fibrosis.

## Additional files

Additional file 1: **Table S1.** Primer sequences for qRT-PCR. (DOCX 18 kb)

Additional file 2: **Figure S1.** mRNA expressions of inflammation cytokines and anti-oxidant molecules in acute and chronic liver disease models by CCl_4_ injections. (A-C), mRNA expression of pro-inflammatory cytokines. (D-I), mRNA expression of anti-oxidant molecules. (**P < 0.05, ***P < 0.005, C + A; CCl_4_ + iHEPs-A, C + B; CCl_4_ + iHEPs-B). (TIF 1549 kb)

## Abbreviations

ALT: Alanine aminotransferase
AST: Aspartate aminotransferase
CAT: Catalase
CCl_4_: Carbon tetrachloride
ESCs: Embryonic stem cells
GST: Glutathion S-transferase
HO-1: Heme oxygenase 1
iHEPs: Induced hepatocyte-like cells
IL-1β: Interleukin-1β
IL-6: Interleukin-6
iPSC: Induced pluripotent stem cells
iPSC-HLCs: iPSC-derived hepatocyte-like cells
MEF: Mouse embryonic fibroblast
MELD: Model for End-stage Liver disease
NQO-1: NADPH dehydrogenase quinone 1
NRF2: Nuclear factor erythroid-derived 2-like 2
SOD-1: Superoxide dismutase 1
TNFα: Tumor necrosis factor α
