# Supplementary material for: The therapeutic potential of induced hepatocyte-like cells generated by direct reprogramming on hepatic fibrosis
Source: Stem Cell Res Ther. 2019 Jan 11;10:21. doi: 10.1186/s13287-018-1127-3 (PMC6330392; doi:10.1186/s13287-018-1127-3)
Supplement: Supplementary file 1 — Table S1. Primer sequences for qRT-PCR. (DOCX 18 kb) [file 13287_2018_1127_MOESM1_ESM.docx]

**Table S1. Primer sequences for qRT-PCR**

| **Name** | **Forward sequence (5’ -> 3’)** | **Reverse sequence (5’->3’)** |
| --- | --- | --- |
| Mouse COL1α1 | GAGCGGAGAGTACTGGATCG | TACTCGAACGGGAATCCATC |
| Mouse TGFβ1 | ATACGCCTGAGTGGCTGTCT | GGTTCATGTCATGGATGGTG |
| Mouse αSMA | ACTGGGACGACATGGAAAAG | AGAGGCATAGAGGGACAGCA |
| Mouse Fibronectin | GAGCTGCACATGTCTTGGGAAC | GGAGCAAATGGCACCGAGATA |
| Mouse TNFα | AGGGTCTGGGCCATAGAACT | CCACCACGCTCTTCTGTCTAC |
| Mouse IL-1β | TGCCACCTTTTGACAGTGATG | ATGTGCTGCTGCGAGATTTG |
| Mouse IL-6 | TAGTCCTTCCTACCCCAATTTCC | TTGGTCCTTAGCCACTCCTTC |
| Mouse NRF2 | TGCCCCTGGAAGTGTCAAACA | CAACAGGGAGGTTAATGATTT |
| Mouse HO-1 | ACAGATGGCGTCACTTCG | TGAGGACCCACTGGAGGA |
| Mouse NQO1 | CTTTAGGGTCGTCTTGGC | CAATCAGGGCTCTTCTCG |
| Mouse CAT | AAATGCTTCAGGGCCGCCTT | GTAGGGACAGTTCACAGGTA |
| Mouse GST | CAAAGCAGGCCATAGACAGGG | AAAAGCGTGAATGGGGCATAC |
| Mouse SOD1 | ccagtgcaggacctcatttt | cacctttgcccaagtcatct |
| Mouse GAPDH | ACTGGCATGGCCTTCCGTGT | ACTGGCATGGCCTTCCGTGT |
