# Supplementary figures and images for: The therapeutic potential of induced hepatocyte-like cells generated by direct reprogramming on hepatic fibrosis
Source: Stem Cell Res Ther. 2019 Jan 11;10:21. doi: 10.1186/s13287-018-1127-3 (PMC6330392; doi:10.1186/s13287-018-1127-3)

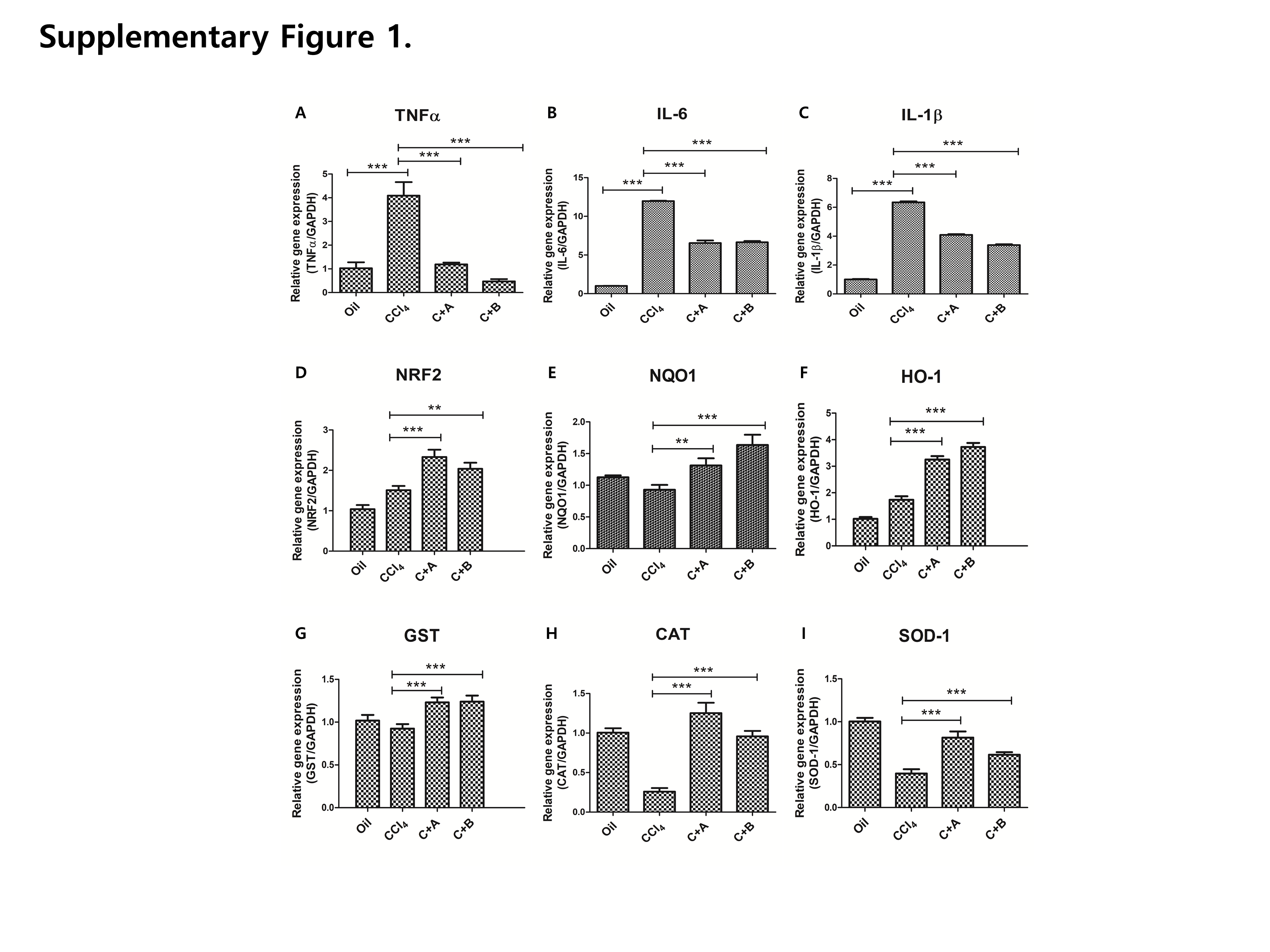

Supplement: Supplementary file 2 — Figure S1. mRNA expressions of inflammation cytokines and anti-oxidant molecules in acute and chronic liver disease models by CCl4 injections. (A-C), mRNA expression of pro-inflammatory cytokines. (D-I), mRNA expression of anti-oxidant molecules. (**P < 0.05, ***P < 0.005, C + A; CCl4 + iHEPs-A, C + B; CCl4 + iHEPs-B). (TIF 1549 kb) [file 13287_2018_1127_MOESM2_ESM.tif]
